# Vertical sonar beam width and scanning behavior of wild belugas (*Delphinapterus leucas*) in West Greenland

**DOI:** 10.1371/journal.pone.0257054

**Published:** 2021-09-09

**Authors:** Marie J. Zahn, Kristin L. Laidre, Peter Stilz, Marianne H. Rasmussen, Jens C. Koblitz

**Affiliations:** 1 School of Aquatic and Fishery Sciences, University of Washington, Seattle, WA, United States of America; 2 Polar Science Center, Applied Physics Laboratory, University of Washington, Seattle, WA, United States of America; 3 Animal Physiology, Institute for Neurobiology, University of Tübingen, Tübingen, Germany; 4 The University of Iceland’s research center in Húsavík, Húsavík, Iceland; 5 Max Planck Institute of Animal Behavior, Konstanz, Germany; 6 Centre for the Advanced Study of Collective Behaviour, University of Konstanz, Konstanz, Germany; 7 Department of Biology, University of Konstanz, Konstanz, Germany; Oregon State University, UNITED STATES

## Abstract

Echolocation signals of wild beluga whales (*Delphinapterus leucas*) were recorded in 2013 using a vertical, linear 16-hydrophone array at two locations in the pack ice of Baffin Bay, West Greenland. Individual whales were localized for 4:42 minutes of 1:04 hours of recordings. Clicks centered on the recording equipment (i.e. on-axis clicks) were isolated to calculate sonar parameters. We report the first sonar beam estimate of *in situ* recordings of wild belugas with an average -3 dB asymmetrical vertical beam width of 5.4°, showing a wider ventral beam. This narrow beam width is consistent with estimates from captive belugas; however, our results indicate that beluga sonar beams may not be symmetrical and may differ in wild and captive contexts. The mean apparent source level for on-axis clicks was 212 dB pp re 1 μPa and whales were shown to vertically scan the array from 120 meters distance. Our findings support the hypothesis that highly directional sonar beams and high source levels are an evolutionary adaptation for Arctic odontocetes to reduce unwanted surface echoes from sea ice (i.e., acoustic clutter) and effectively navigate through leads in the pack ice (e.g., find breathing holes). These results provide the first baseline beluga sonar metrics from free-ranging animals using a hydrophone array and are important for acoustic programs throughout the Arctic, particularly for acoustic classification between belugas and narwhals (*Monodon monoceros*).

## Introduction

Arctic cetacean species are faced with a growing threat of underwater noise pollution as vessel traffic is expected to increase with rising temperatures and ice-free conditions [1–3]. The Arctic is warming at a dramatic rate of two to three times the global mean with expected ice-free summers between 2030 to 2055 [4–7]. Endemic Arctic cetacean species have evolved to use sound to locate prey and communicate with each other in ice-dominant conditions. Man-made sounds such as engines, propellers, and air guns produce considerable noise underwater, interfering with the ability for cetaceans to sense their surroundings and find food [2, 8, 9]. The Northwest Passage and Northern Sea Route that are increasingly ice-free overlap spatially with key foraging areas and migration routes for these Arctic cetacean species [1, 2]. As the open-water season lengthens, human expansion in the Arctic through commercial shipping, fishing, and oil and gas exploration poses substantial risks to Arctic marine mammals in the form of vessel strikes, acoustic disturbance, and exposure to spilled or leaked oil and other harmful chemicals [1, 2, 10]. With these anticipated changes, scientists have an opportunity to inform management of human activities in the Arctic to minimize negative impacts on cetaceans through strategies such as restricting ship speed and rerouting vessels to avoid key habitat areas [1, 10–12].

Beluga whales (*Delphinapterus leucas*) are one of only two endemic Arctic odontocetes—along with the narwhal (*Monodon monoceros*)—that occupy the Arctic year-round. Unlike the narwhal, belugas have a circumpolar distribution. They are comprised of discrete subpopulations both in the Arctic and sub-Arctic [1, 13–15]. Given their large distribution and apex trophic position, belugas serve as an important ocean sentinel in a changing Arctic. While the eventual impacts of climate-induced habitat change on belugas remains unknown, monitoring their annual distribution and changes thereof are key to determine their responses and adaptations. Passive acoustic monitoring (PAM) is the most appropriate approach to study cetacean distributions, particularly for regions difficult to access such as the Arctic. It has been used to estimate range, seasonality, and population density of cetaceans [16, 17], all of which are important considerations for managers to minimize spatial overlap and potentially harmful impacts of the developing shipping industry in the Arctic.

Affectionately named the “canaries of the sea,” belugas are social and vociferous whales with highly variable acoustic profiles that use echolocation as a primary sense to target prey and spatially orient. They produce pulsed calls, combined calls, whistles, and echolocation clicks within broad frequency ranges from roughly 100 Hz to 20 kHz with some echolocation clicks reaching 120 kHz and higher [18–20]. Despite the extensive work done on beluga acoustics, only three publications present information on echolocation of free-ranging animals [20–22] with only one providing results on echolocation click parameters [22]. Most studies focus on non-echolocation vocalizations [19, 20, 23, 24], investigate the echolocation capabilities of animals in captivity [18, 25–31], or use recorded echolocation clicks as a proxy for animal presence during PAM [32–36]. Beluga echolocation occurs within a wide spectral range of 30 to 120 kHz where most of the click energy is concentrated within 30–50 kHz and can change depending on the ambient noise in their environment [18, 20, 22]. Still, the spectral characteristics of free-ranging beluga echolocation have not been examined at a high resolution.

The acoustic field of view of an echolocating animal, referred to as its sonar beam, is controlled by the properties of functional range (intensity) and angular coverage (directionality) of the emitted clicks [37–39]. By producing short, high-frequency signals, the animal ensonifies a three-dimensional area and listens for echoes to interpret its surroundings and locate prey. The source level (SL) is known as the intensity of the signal recorded on the acoustic axis of the beam at a standardized 1 m distance from the source [40, 41] and can conveniently be quantified as the peak-to-peak (pp) sound pressure level for stereotypical signals like toothed whale echolocation clicks. Echolocation clicks are highly directional such that the signal intensity rapidly decreases with increasing angle away from the acoustic axis [38, 42]. Thus, directionality refers to the angular width in degrees (°) of the biosonar beam and is measured at the angle where the click intensity has decreased by half (-3 dB) relative to the on-axis click intensity.

There is evidence demonstrating toothed whales have active control over their sonar beam by adjusting output levels [18, 43], beam width [37, 44, 45], and the direction of the beam known as their acoustic gaze [45–47]. Further, the biosonar beam is not only characterized by the signal itself, but the morphology of the sound generator and size of the animal [48]. However, reported beam width measurements from toothed whales converge around a narrow 5–14°, leading Jensen et al. [48] to conclude that observed inverse frequency scaling where larger animals emit lower frequencies and smaller animals emit higher frequencies is driven by the evolutionary pressure to maintain a narrow acoustic field of view. A narrow beam facilitates precise sensory analysis of the animal’s environment by reducing unwanted echoes and clutter [46, 48]. Yet, the advantages of a narrow beam come with the cost of a small ensonified volume which can increase the risk of missed detections and reduce the animal’s sensory awareness. Scanning behavior, where an animal surveys the surrounding area by actively moving its sonar beam at various angles, may mitigate the costs associated with a narrow beam by increasing search volume and aiding in the localization of live prey [45, 49]. As the only Arctic odontocetes, the narwhal and beluga have evolved in a unique marine environment characterized by sea ice with similar evolutionary pressures shaping their acoustic profiles. Koblitz et al. [46] reported that the narwhal has the most directional sonar beam among all odontocetes with a 5° beam width. For the beluga, Au et al. [25] conducted a captive experiment and determined the beluga beam width to be 6.5°. However, to date no estimate of beam width has been made for free-ranging belugas. Given the potential for variation in biosonar properties based on context, it is unknown whether belugas share an exceptionally narrow beam width like the narwhal in the wild.

Arguably the most important parameters that characterize whale biosonar systems are beam width, SL, and inter-click-interval as they provide information about how echolocation beams have evolved and how whales can be effectively monitored using passive acoustics [38, 41]. Yet, these metrics are especially difficult to measure in the wild because animal position estimates and methods to isolate on-axis clicks are required [38]. Here, we use data from a 16-channel vertical hydrophone array to determine baseline acoustic parameters including vertical beam width and SL of *in situ* beluga echolocation. Our results represent a unique dataset of beluga echolocation from a wild context, filling critical data gaps for the understudied population in Baffin Bay, West Greenland. We discuss how our findings provide foundational data useful for PAM programs and contribute to the broad understanding of beluga acoustic ecology, including how they have evolved to use sound to navigate, forage, and communicate with one another in an ice-dominant environment.

## Materials and methods

### Study region and acoustic recordings

**Fig 1:**
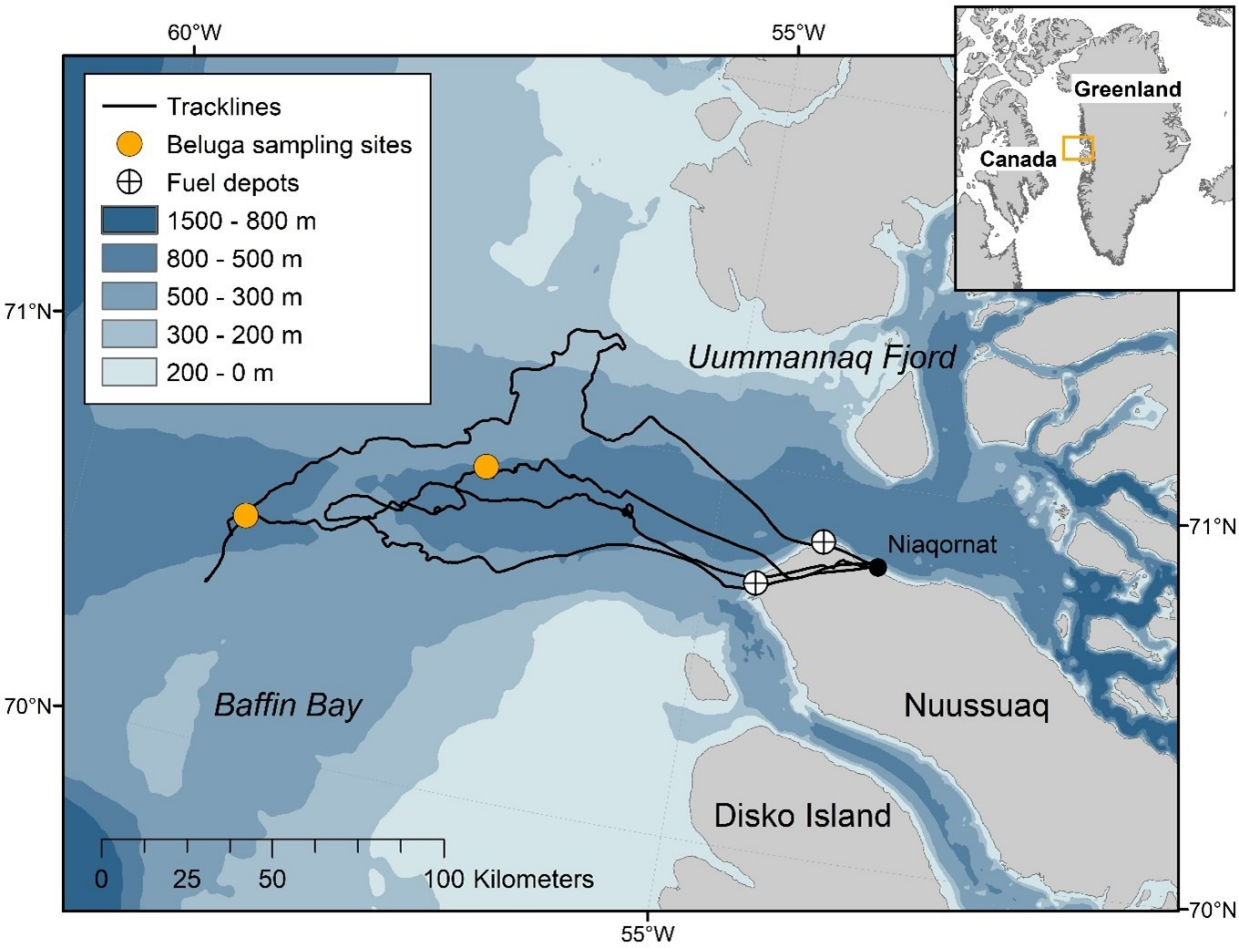
Map of study region in Baffin Bay, West Greenland. Includes track lines of search effort, fuel depots, and sampling locations on March 25 and 31^st^, 2013.

During March 2013, helicopter-based surveys were conducted out of Niaqornat, West Greenland from an Air Greenland AS350. Suitable weather permitted seven flying days between 21 and 31 March and searches for whales occurred 100 to 150 km offshore in leads and cracks of pack ice >98% concentration (Fig 1). The primary objective of the study was to locate and record narwhals [46, 50], however, on two occasions pods of about 6 and 30 belugas were recorded opportunistically (Fig 1). Beluga whales were observed from the air and then sea ice conditions and weather were assessed for landing. As soon as possible after landing, a hydrophone array was deployed at the edge of a lead positioned in a vertical, linear orientation. Belugas were visible the entire time during the recording period and no narwhals were in the vicinity.

The array was composed of 16 individual Reson TC4013-5 hydrophones with a sensitivity of -215 dB +/- 2dB re 1V/μPa and flat (+/-2 dB) frequency response between 1–150 kHz. Each hydrophone was spaced 1 m apart on a 2 mm diameter line with the topmost hydrophone at 3 m below the surface, the lowest at 18 m, and a 4 kg weight was tied to the bottom to maintain verticality. A custom 16-channel amplifier was used to amplify hydrophone signals by 35 dB; the hydrophones functioned as a low pass filter (150 kHz, 1 pole) and no high pass filter was applied. The recordings were converted from analog to digital with a sampling rate of 500 kHz and 16-bit resolution using two eight-channel National Instruments PXI-6123 A/D converters (voltage input range +/-10 V). A custom software MALTA (Microphone Array Localization Tool for Animals) by CAE Software & Systems was used to visualize recordings in real-time of multiple receivers. Recordings were partitioned, loss-less, in 5-second long WAV files as a safeguard against file corruption and for ease in data processing and analysis. Clipping level of the recording system was at 206 dB pp re 1 μPa at 100 kHz [46]. All 16 hydrophones were calibrated prior to deployment, and the resulting frequency response of each receiver determined. Localization accuracy was determined in the field using playbacks of recorded clicks at known distances from the array [46].

### Localization and assignment of clicks to individuals

Recordings were visually inspected for the presence of beluga echolocation and then used for localization analysis. Clicks were detected using channel 10 just below the center of the array with a signal to noise ratio (SNR) greater than 12 dB. A time window of -5 and +6 ms (5500 samples) was extracted around each click detection for each channel and used for downstream analyses. The position of the individual at the time of click emission was calculated using the time of arrival difference (TOAD). Due to the vertical, linear organization of the hydrophones, the distance and depth of the whale were estimated but the direction in the horizontal plane could not be determined. Cross correlations of all 120 possible receiver pairs were calculated and the channel identified to have best match with other receivers was used as a reference. This reference channel was then used to compute TOADs between the 15 remaining channels and the beluga source position was determined based on a least square error optimization [51]. For best localization accuracy, up to eight channels were excluded from localization calculations for instances where localizations based on this receiver substantially differed from the remaining channel combinations. In most cases, position estimates were based on all 16 channels but a minimum of eight channels were used. To confirm localization accuracy of the least square numerical solution for each click, the calculated source position was plotted over 28–120 hyperbolas from the receiver pair TOADs considered [38, 51].

Manual assignment of clicks to echolocation sequences were based on the continuity between localized estimates of distance and depth, the sound pressure level recorded on each receiver—termed the received level (RL)—and inter-click interval (ICI). Clicks emitted beyond 150 m from the array were not considered due to high localization error at distances greater than ten times the array aperture (15 m). Inconsistent positions, ICI, and RL values indicated overlapping click trains from more than one individual. Using these patterns, clicks were assigned to individuals as separate tracks. Since multiple, successive echolocation sequences may be produced by the same whale, we used a conservative approach for identifying individuals using criteria of spatial and temporal proximity. Click sequences were assumed to originate from the same individual if 1) the whale’s track line was consistently approaching the array, and 2) click sequences were not separated by more than 10 seconds or 10 meters in depth or distance. Localized clicks were isolated, bandpass filtered (1 kHz 4^th^ order high-pass and 240 kHz 4^th^ order low-pass), and sensitivity for each receiver incorporated to calculate source properties.

### Sonar parameter calculations and on-axis click criteria

Peak frequency (kHz), -3 dB and -10 dB bandwidth (kHz), -10 dB click duration (μs), apparent source level (ASL), ventral and dorsal -3 dB beam width (°), and the transmission beam directivity index (DI; dB) were calculated for all localized clicks. Peak frequency was defined as the frequency of maximum amplitude in the spectrum [38]. The -3 dB and -10 dB bandwidth (kHz) were calculated as measures of the spectral variance around the centroid frequency of the spectrum, and the -10 dB click duration (μs) was determined using -10 dB spectral bounds relative to the peak of the waveform envelope [38]. The ASL was computed over the -10 dB click duration using the sonar equation and assumed geometric spreading and signal attenuation:

ASL=RL+TL

TL=20log(r)+αr

where *RL* is received level, or sound pressure level recorded on each receiver, *TL* is transmission loss, α is the absorption (0.03 dB/m at 100 kHz) [52], and *r* is the range, or derived distance of 1 m in front of the animal to the receiver. Following Møhl et al. [40], the peak-peak apparent source level (ASL_pp_) of each click was determined by the peak-peak measurement of its Hilbert transformation re 1 μPa and back-calculated to 1 m distance in front of the beluga. Given echolocation clicks are very short and broadband, it is possible that the actual peak amplitude of the signal is missed when sampling at 500 kHz. However, the amplitude of the Hilbert envelope effectively approximates the absolute analog amplitude despite sampling limitations. The root mean square apparent source level (ASL_rms_) measured at 1 m was defined by the RL_rms_ pressure over the -10 dB click duration. The energy flux density source level at 1 m was the integrated energy of the signal over a -10 dB duration and was calculated by ASL_rms_+10log(-10 dB click duration) [53].

Given the vertical orientation of the linear hydrophone array, only the vertical beam width was calculated. To calculate the vertical -3 dB beam width, vertical beam patterns based on measured RL at each receiver were merged and aligned at the receiver of maximal intensity, identified as the center of the beam. Using the approximated -3 dB beam width, the sonar beam directivity index (DI) was then calculated following the approximation by Zimmer et al. [54]: Θ = 185° x 10^(-DI/20)^, where Θ denotes -3dB beam width.

**Fig 2:**
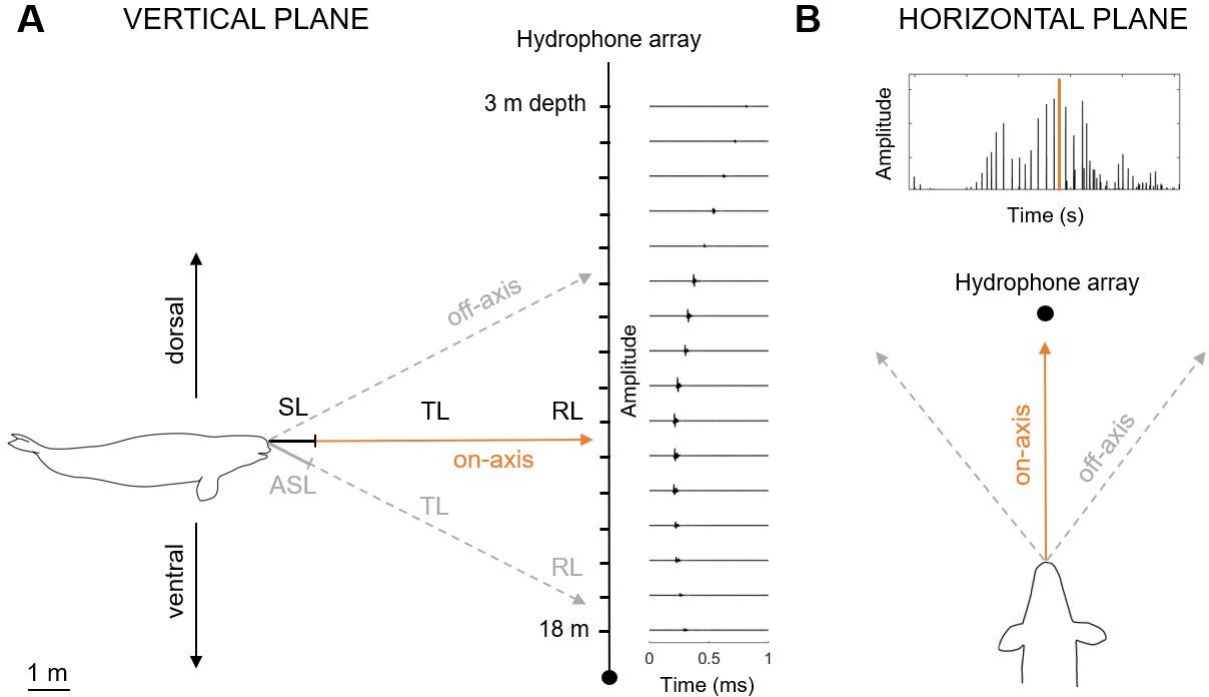
Schematic showing experimental setup and spatial criterion for on-axis click selection in the (A) vertical and (B) horizontal planes. The radiation pattern of an echolocation click is shown where the passive sonar equation was applied. The source level (SL) is the sound pressure level calculated from the received level (RL) and transmission loss (TL) on the acoustic axis; apparent source level (ASL) is the sound pressure level calculated for off-axis clicks/clicks of unknown recording aspect. Vertical on-axis clicks were isolated when the whale’s acoustic axis (i.e. the maximal intensity of the whale’s beam) was vertically centered on the array which was determined by -3dB beam width calculations. For each scanning sequence (i.e. click train with first ascending then descending amplitudes), the highest intensity click was isolated as a horizontal on-axis click where the whale was pointing its beam towards the array (not to the left or right), assuming constant SL.

Echolocation clicks are highly directional where signals received oblique to the animal’s acoustic axis are distorted [38, 42]. Precise derivation of acoustic source properties thus depends on the isolation of clicks recorded as close to the whale’s acoustic axis as possible, herein referred to as on-axis clicks [38]. On-axis clicks are characterized by the condition where the maximal intensity of the whale’s beam is aimed directly towards the hydrophone array in both the vertical (up and down) and horizontal (left and right) dimensions (Fig 2). Following previous studies [46, 55–58], we selected on-axis clicks based on specific criteria. On-axis clicks in the vertical plane were recorded clicks where the whale’s beam was centered between the top and bottom receivers (Fig 2A). If the whale’s apparent beam maximum was detected at one of the outermost receivers, it was assumed that the beam axis was directed outside of the array, and at best, only part of the ventral or dorsal beam was captured. Therefore, clicks were identified as vertically on-axis when both the ventral and dorsal -3 dB beam width could be calculated within the array. Similarly, on-axis clicks in the horizontal plane were recorded clicks where the whale’s beam is directed straight towards the receivers, not pointing to the left or right of the array (Fig 2B). Horizontal on-axis clicks were isolated by selecting the highest amplitude click that is part of a scan (i.e. click train where amplitude first increases then decreases) with the assumption that the animal maintains a constant source level and is scanning the array in both vertical and horizontal planes. All other clicks were defined as being off-axis clicks. While up to eight channels were excluded for localization calculations, recordings from all 16 receivers were used for beam width estimates.

Given the on-axis characteristics defined above, we selected clicks for final sonar parameter values when they fulfilled the following criteria: (1) localized within 120 m of the array; (2) part of a click train defined by a sequence of more than 10 clicks; (3) the maximal intensity was not recorded at one of the outermost receivers; (4) both the ventral and dorsal -3 dB beam width were calculated; (5) part of a scan; (6) highest amplitude click in the scan; (7) one click selected per track to avoid pseudo-replication of individuals. For comparison, sonar parameters were calculated for two subsets of data: (1) on-axis clicks in only the vertical plane (criteria 1–4), and (2) on-axis clicks in both the vertical and horizontal planes (criteria 1–7). Using these two datasets, the degree of click parameter distortion when introducing horizontal off-axis clicks was evaluated. Finally, clicks where the maximal intensity was recorded at the top- or bottom-most receiver were removed for a third subset of data that included only “non-edge clicks,” where edge refers to the outermost receivers in the array (criteria 1–3). Non-edge clicks were used to examine angular variation in beluga spectra and waveforms to see how beluga signals change with increasing angle (2 – 20°) away from the whale’s acoustic axis (0°).

Once clicks were isolated for on-axis clicks in the vertical plane and on-axis clicks in both the vertical and horizontal planes, final calculations of mean sonar parameter values were determined. To account for any minor hydrophone sensitivity fluctuations not explained in calibrations, all vertical beam width measurements were interpolated to a resolution of 0.5° by applying a flat smoothing function where 5 smoothing points were used for a moving average over 2°. The 2° width corresponded to the average localized source distances and sufficiently addressed any local peak artifacts from individual hydrophones. The final -3 dB beam width was determined by averaging individual -3 dB beam width values from on-axis clicks. The mean vertical beam pattern for all aligned on-axis clicks was smoothed (width of 2°) and normalized at their center to zero. While the angle of the emitted click to the acoustic axis was unknown, we expected our ASL estimates to be close to the true source level by following the criteria outlined above for isolating on-axis clicks.

After on-axis clicks were selected, inter-click intervals (ICIs) were calculated for several conditions. ICIs are defined as the time interval in milliseconds between successive clicks. Median ICI values were calculated for: 1) the interval preceding each selected on-axis click (pre-click), 2) the interval proceeding each on-axis click (post-click), 3) a pooled sample of pre- and post-click intervals, and 4) intervals between all clicks from same localized click sequences, or tracks, that on-axis clicks were selected from.

## Results

**Fig 3:**
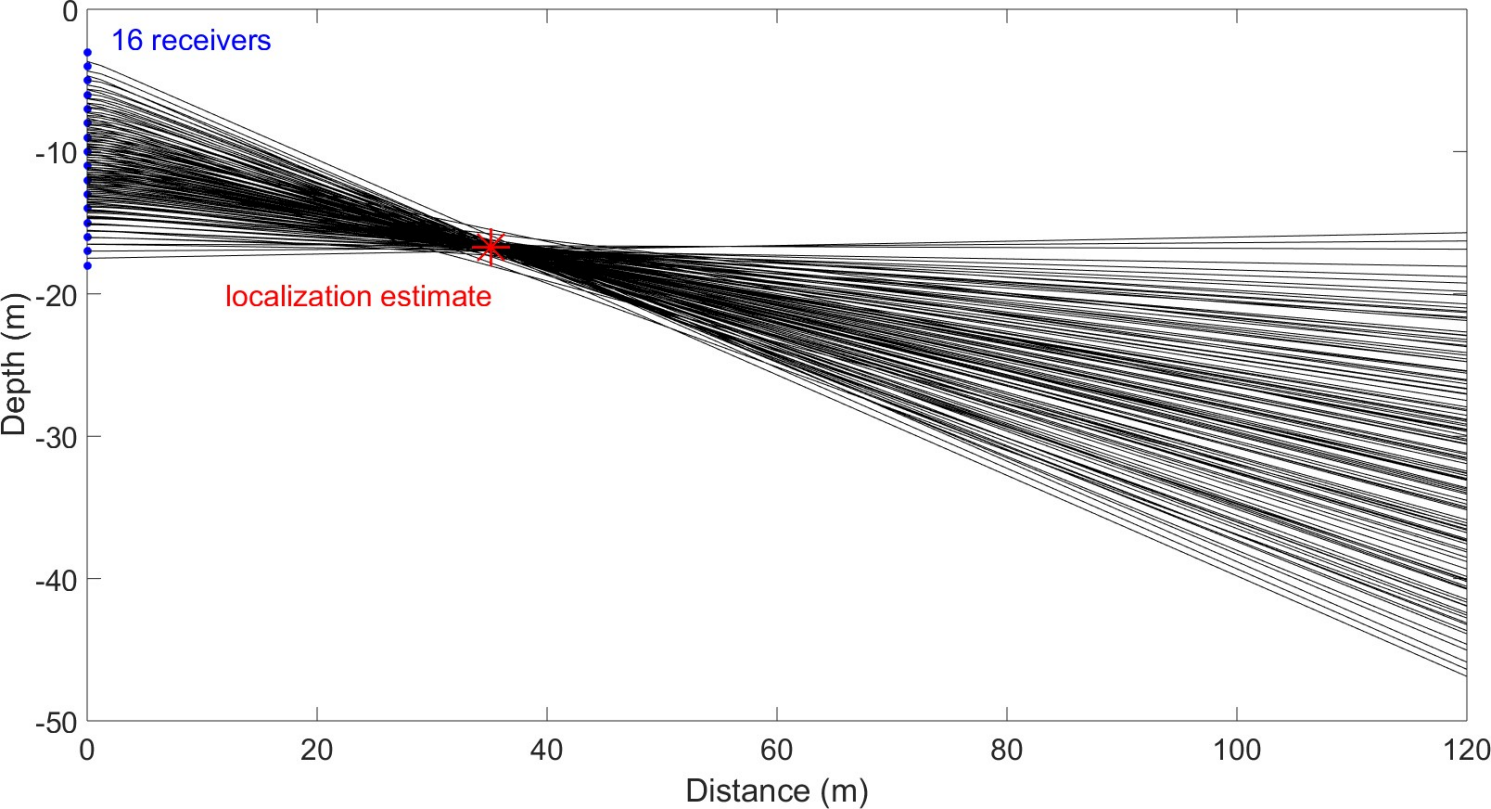
Hyperbolae for each of the 120 hydrophone pairs for a single click. Each blue dot on the y-axis indicates a hydrophone in the vertical array spaced 1 m apart. The red star demarcates the localization estimate in depth and distance from the array based on the least squares model. The hyperbolae intersect at the location of the analytical solution.

**Fig 4:**
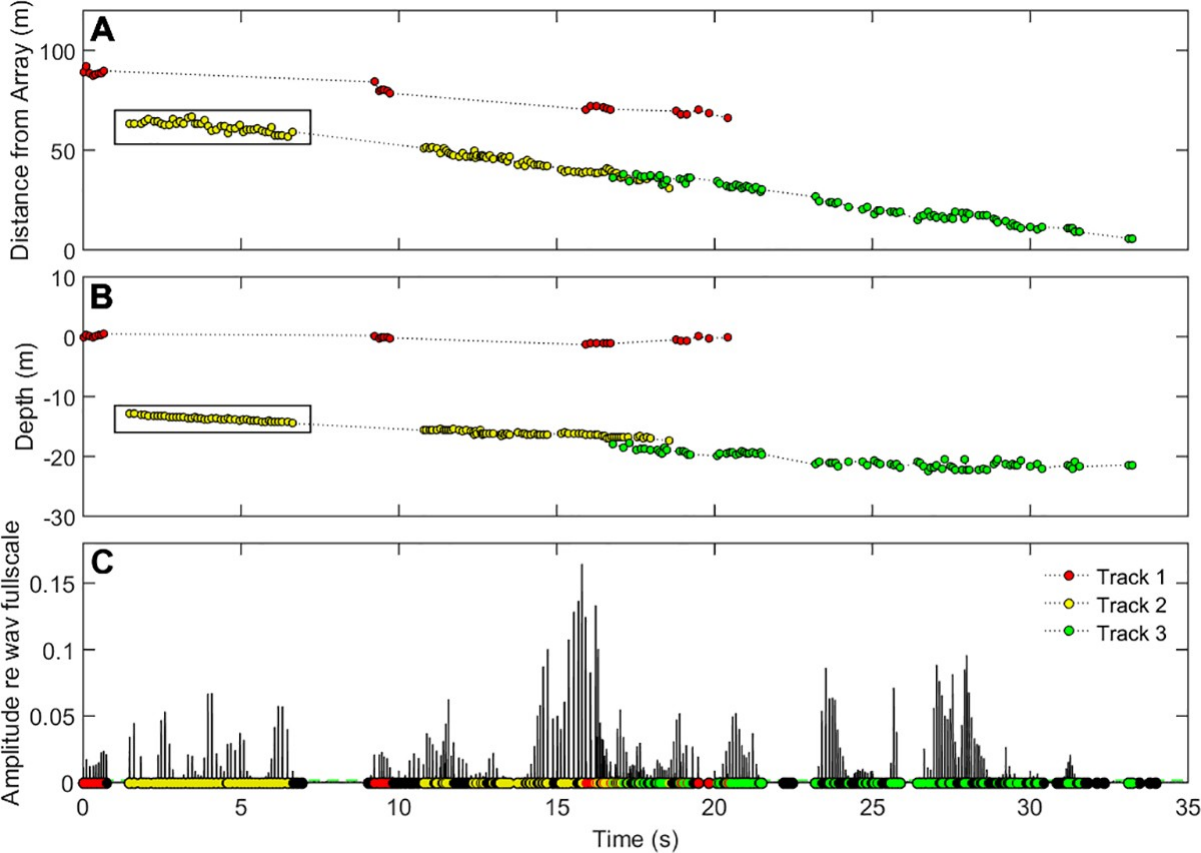
Echolocation localization tracklines assigned for at least three individual belugas. (A) localized distance from the array, (B) localized depth, and (C) Hilbert transform of the received amplitude for channel 10 recordings used for click detections. The black box indicates the click sequence used for Fig 7. Red circles are track 1, yellow circles track 2, and green circles track 3. Spatial information separates the individual shown in red from the individuals in yellow and green. Patterns in ICI, RL, and localized distance and depth allow for separation of the individuals assigned in yellow and green.

Beluga recordings were made at two locations on March 25^th^ and 31^st^ 2013 for a total of 42:40 and 20:45 minutes, respectively. Beluga data reported in this study were from one site and individual whales were localized for 4:42 minutes. Each location was validated by comparing the least square localization estimate with hyperbolae for each hydrophone pairwise solution (see Fig 3). Out of 1,876 total clicks detected above a threshold of 146 dB pp re 1 μPa, 672 clicks were assigned to individuals in 17 separate tracks (see Fig 4). 133 clicks were isolated as on-axis in only the vertical plane and 12 clicks were selected as on-axis in both the vertical and horizontal planes. All on-axis clicks originated from 12 of the 17 total separate tracks and were used for sonar parameter calculations. For angular variation analysis, 351 clicks were isolated as being non-edge clicks where the maximal intensity of the click was not recorded at one of the outermost receivers. We estimate at minimum three to six individual belugas produced these tracks.

### Beluga vertical beam width and sonar parameters

**Fig 5:**
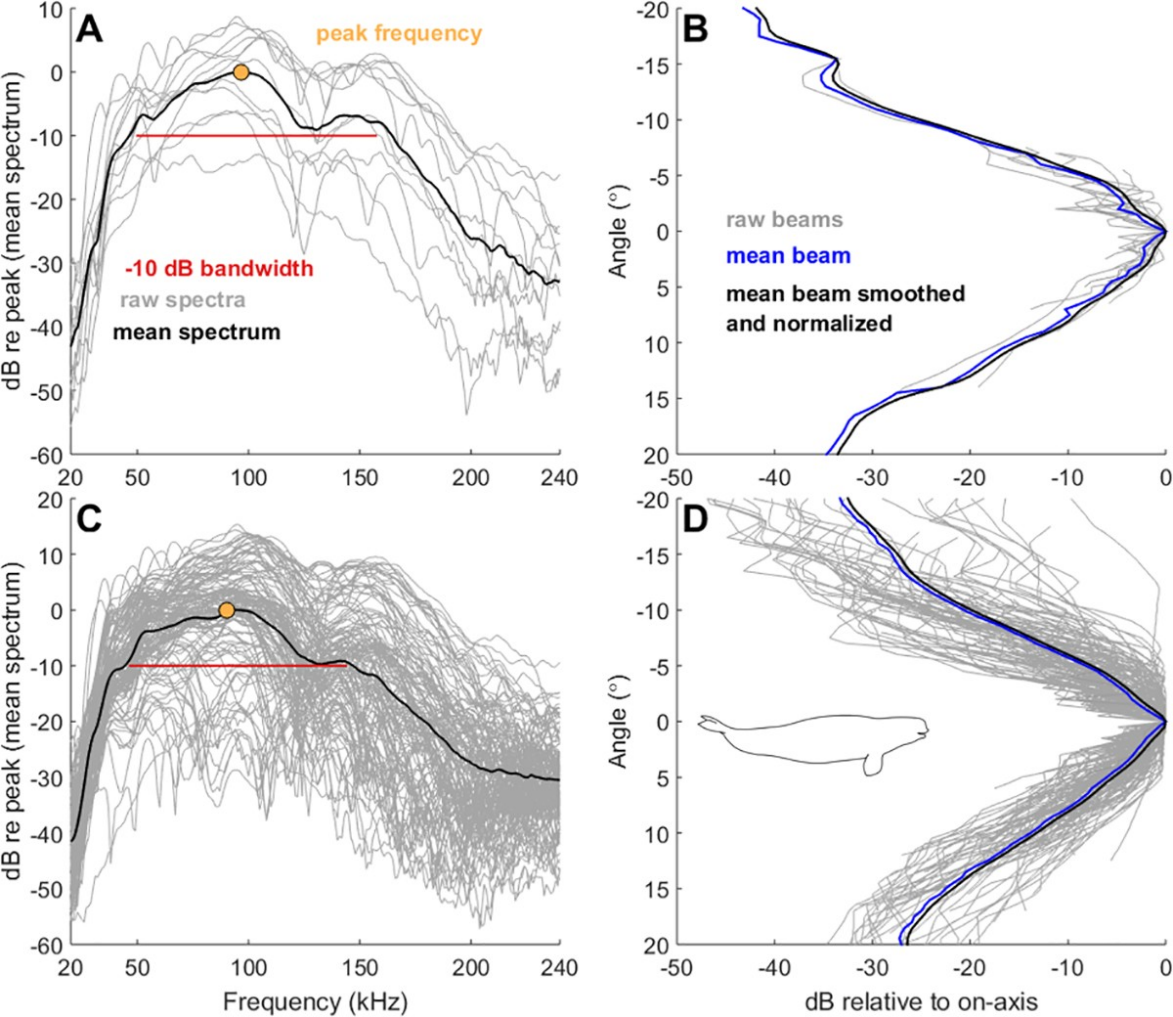
Beluga echolocation click spectra and beam pattern for on-axis clicks. (A) Beluga spectra and (B) vertical beam pattern for 12 on-axis clicks in both the vertical and horizontal planes. (C) Beluga spectra and (D) vertical beam pattern for 133 on-axis clicks in only the vertical plane. Spectra in (A) and (C) show raw spectra in gray, mean spectrum in black, peak frequency with a yellow point, and -10 dB bandwidth in red. Vertical beam patterns in (B) and (D) show raw beam patterns in gray, mean beam pattern in black, and the smoothed and normalized beam pattern in blue; negative angle values correspond to the dorsal vertical beam and positive values refer to the ventral beam.

Mean beluga sonar parameter values were similar between on-axis clicks in the vertical and horizontal planes and clicks on-axis only in the vertical plane (Table 1). A total of 133 clicks were on-axis in the vertical plane where both the ventral and dorsal -3 dB beam width could be determined, indicating that the echolocation beam was vertically centered on the array. Among these, 12 clicks were determined to be on-axis in both the vertical and horizontal planes using the criteria outlined above. Mean beam width values for on-axis clicks in the vertical plane were larger (i.e., wider) and had larger ranges than clicks on-axis in both planes. Sonar parameter values for on-axis clicks in the vertical plane had greater variation than on-axis clicks in the vertical and horizontal planes (Table 1; Fig 5). All sonar parameter estimates are summarized in Tables 1 and 2.

**Table 1:** Mean (± s.d.) and range of wild beluga sonar parameters calculated for all on-axis clicks with values from previous work included for comparison.

	On-axis in horizontal and vertical planes		On-axis in vertical plane		Previous work	
	(*n* = 12)		(*n* = 133)			
Parameter	mean ± s.d.	range	mean ± s.d.	range	mean ± s.d.	range
-3 dB beam width (°)	5.4 ± 1.2	3.8–8.1	6.4 ± 2.4	2.4–18.3	6.5^‡^	
-3 dB dorsal beam width (°)	2.0 ± 1.0*	0.6–3.6	2.9 ± 2.0*	0.5–14.6		
-3 dB ventral beam width (°)	3.4 ± 1.0*	1.3–4.8	3.5 ± 1.5*	1.1–8.5		
DI (dB)	30.9 ± 1.8	27.2–33.7	29.8 ± 3.3	20.1–37.7	32.1^‡^	
ASL_pp_ (dB re 1 μPa)	212 ± 6	198–219	206 ± 8	184–219	198 ± 5–206 ± 6^†^;	NA– 222^†^;
					218 ± 5^‡^;	206–218^‡^;
					164 ± 10^§^	150–184^§^
ASL_rms_ (dB re 1 μPa)	206 ± 6	193–214	199 ± 9	175–214		
ASL_EFD_ (dB re 1 μPa^2^s)	158 ± 6	144–164	152 ± 8	131–164	145 ± 5–149 ± 5^†^	NA– 165^†^
Peak frequency (kHz)	96.9 ± 7.4	88.0–110.0	90.1 ± 20.7	52.0–159.0	~55, ~105^†^;	40–60, 100–120^†^;
					107, 113^‡^	10–54^§^;
					40 ± 6^§^;	32–90, 40–120^††^
					73^††^	
-3 dB bandwidth (kHz)	39.8 ± 19.6	19.0–62.0	45.4 ± 18.1	15.0–115.0	~23, ~37^†^;	15–30, 30–65^†^;
					13 ± 4^§^	6–38^§^
-10 dB bandwidth (kHz)	107.3 ± 21.1	68.0–137.0	97.2 ± 23.1	42.0–152.0	29 ± 10^§^	12–53^§^
Duration_-10 dB_ (μs)	14.5 ± 6.0	8.0–26.0	19.4 ± 8.6	– 60.0	163 ± 152^§^	35–1470^§^
Localized depth (m)	19.7 ± 16.0	0.2–44.0	18.2 ± 8.1	0.2–44.3		
Localized distance (m)	56.7 ± 32.3	13.4–99.9	37.9 ± 24.3	5.8–99.9		
Localized range (m)	61.1 ± 28.8	15.6–100.4	39.9 ± 23.3	7.3–100.4		

Subscript “pp” is peak-peak, “rms’ is root-mean-square, and “EFD” is energy flux density. An asterisk (*) indicates significance (*p* < 0.05). References cited include Au et al. [18]^†^, Au et al. [25]^‡^, Roy et al. [22]^§^, Castellote et al. [33]^††^.

**Table 2:** Inter-click interval (ICI) values for on-axis clicks.

	On-axis in horizontal and vertical planes				On-axis in vertical plane			
Interval	median	Q_1_	Q_3_	*n*	median	Q_1_	Q_3_	*n*
All tracks	97.4	76.8	141.1	552	97.4	76.8	141.1	552
Pre-click	89.7	78.7	122.4	12	96.0	77.2	139.2	133
Post-click	108.7	84.2	147.0	12	99.0	77.5	154.6	133
Pre + post	95.0	78.7	133.4	24	105.4	78.2	154.2	166

Median ICI values were calculated for all clicks from same the click sequence, or track, as the selected on-axis clicks (all tracks), before the on-axis click (pre-click), after the on-axis click (post-click), and pooled pre- and post- intervals (pre + post). 25% and 75% quantiles (Q_1_ and Q_3_, respectively) and sample sizes of clicks considered (*n*) are included. All reported values are in milliseconds.

**Fig 6:**
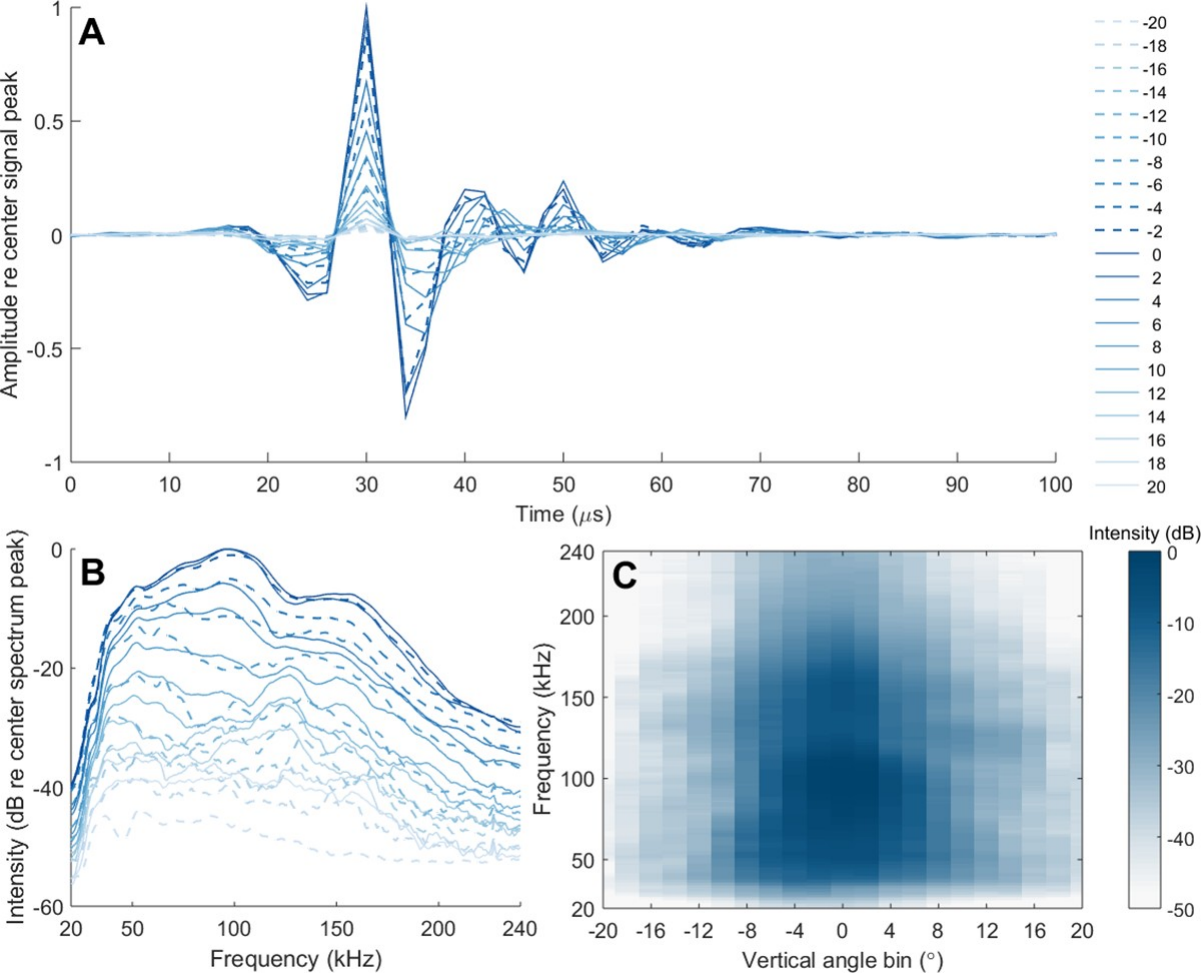
Mean angular variation for 351 non-edge clicks for beluga echolocation click (A) waveforms and (B, C) spectra. For all non-edge clicks selected where the RL of maximal intensity was not recorded at one of the outermost receivers, signals were received at various angles along the vertical hydrophone array where the center of the beam was at 0°. Angle bins between -20 to 20° (negative is dorsal; positive is ventral) show signal differences measured farther from the acoustic axis. The heatmap (C) in dB re center (0°) spectrum peak shows angle bin (°) and frequency ranges for the highest (dark blue) and lowest (white) spectral energy.

A two-lobed spectral pattern was observed for mean beluga spectra (Fig 5A and 5C). The peak frequency for on-axis clicks in both spatial planes was 96.9 ± 7.4 (Table 1; Fig 5A and 5C). A spectral notch occurred at 130 kHz with a secondary peak at 150 kHz. This pattern was more pronounced in the 12 clicks that were on-axis in both planes (Fig 5A) but still evident for clicks that were on-axis in only the vertical plane (Fig 5C). The beluga echolocation mean waveform, consistent with all Delphinidae click shapes, showed an initial pressure increase followed by a strong pressure decrease and second pressure increase (Fig 6A). For all 351 non-edge clicks, signals measured by individual hydrophones away from the acoustic axis (i.e., maximal RL) were averaged in 2° wide angle bins relative to the central axis (0°) and plotted to show angular variation in beluga spectra and waveforms (Fig 6). The signal was increasingly distorted and diminished with increasing off-axis angle (Fig 6). The -3 dB beam width was 5.4 ± 1.2°, ranging between 3.8–8.1° and the ASL_pp_ was 212 ± 6 dB re 1 μPa, ranging between 198–219 dB (Table 1; Fig 5B and 5D). The ventral beam width was significantly wider than the dorsal beam width for on-axis clicks in the vertical plane (Paired *t*-test: *p* = 0.015) and on-axis clicks in both planes (Paired *t*-test: *p* = 0.010). ICIs were approximately 100 ms for selected on-axis clicks and associated click sequences (Table 2).

### Scanning behavior

**Fig 7:**
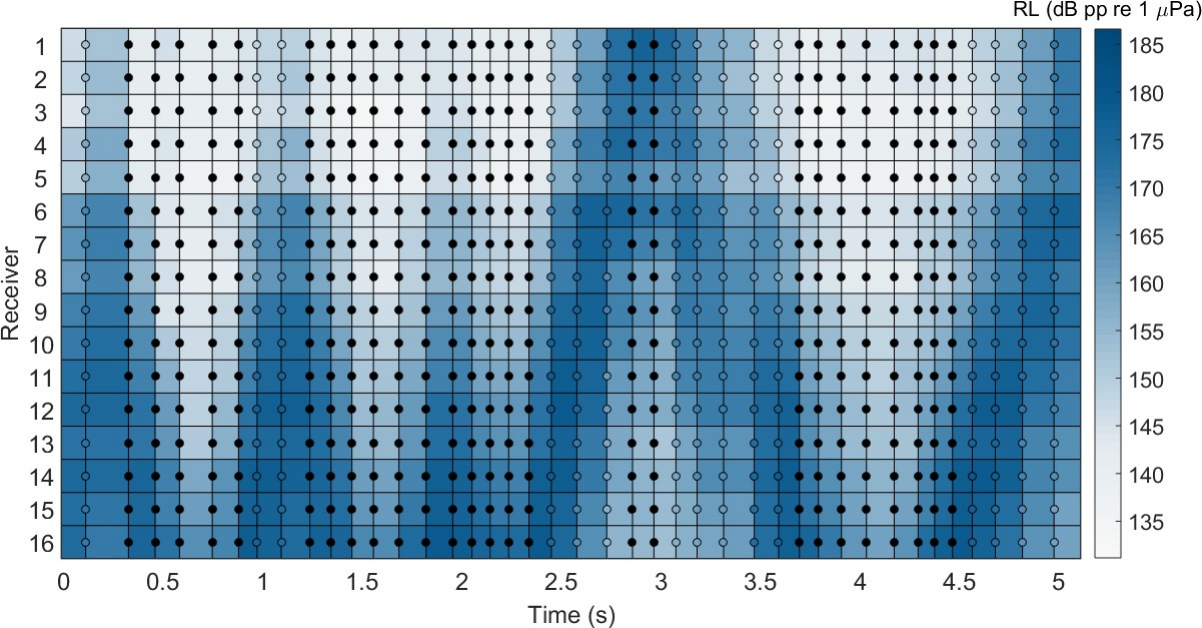
Vertical scanning of beluga sonar beam. Distribution of the received level (RL; represented via color spectrum) for each hydrophone for 41 clicks in 5 seconds, demonstrating vertical scanning of the array. Filled circles signify clicks where the hydrophone with the maximum RL was received by one of the outermost receivers and therefore was directed towards the edge of the array.

Shifts in the acoustic gaze of belugas to probe their environment and increase their search volume—a behavior referred to as scanning—was observed in the vertical plane. Vertical scanning was identified by sequential changes in the maximal RL on individual receivers throughout a click train revealing the upward and downward movement of their sonar beam over the array (Fig 7). All recordings used for analysis where individuals were localized between 10 and 120 meters distance from the array demonstrated this behavior. The maximum scanning angle for consecutive clicks for all analyzed click sequences containing on-axis clicks was 9°.

## Discussion

Despite significant ranges in body size, SL, and sonar frequency among odontocetes, toothed whale beam width remains strikingly consistent between 5–14°, suggesting there is selective pressure for long-range detection and spatial acoustic filtering to reduce unwanted echoes [48]. Beam width and SL are defining characteristics of echolocation that provide insight into the evolution of biosonar systems and key information to biologists who employ passive acoustics for odontocete research and management [38]. Here, we present the first free-ranging beluga vertical beam width estimate of 5.4° and mean ASL of 212 dB pp re 1 μPa with concomitant vertical scanning behavior (i.e., adjustments in acoustic gaze) across all localized sequences. Beluga clicks with high directionality and intensity allow for spatial filtering and a longer detection range while scanning increases acoustic spatial coverage. In the acoustically complex environment of the Arctic, these properties are likely eco-evolutionary adaptations for belugas to reduce clutter and effectively navigate, particularly in the winter as they search for openings in the pack ice.

### Narrow sonar beam width and high source level

The majority of existing knowledge of beluga echolocation comes from experimental work where captive whales were held stationary with a hoop or bite plate to maintain a constant distance and angle to the hydrophone [18, 25–31]. No study has been conducted to measure beluga sonar beam width since the captive experiment completed by Au et al. [25] where they determined the mean 3-dB vertical and horizontal sonar beam width to be a symmetrical 6.5°. Our results showing a vertical beam width of 5.4° (DI = 30.9 dB) confirm the narrow acoustic field of view of belugas, being 1.1° narrower than the estimate made by Au et al. [25]. Au et al. [18] demonstrated how beluga biosonar changes in intensity and frequency for two environments with different ambient noise levels, an indication for the flexibility in echolocation behavior. The minor difference in vertical beam width determined in this study compared to Au et al. [25] may indicate individual or population-specific variation, environment-driven adaptation, or methodological differences.

Being that toothed whale biosonar beam width ranges between 5–14°, the beluga beam width is one of the most narrow among odontocetes, second only to the narwhal [46]. The sperm whale (*Physeter macrocephalus*), Risso’s dolphin (*Grampus griseus*), and bottlenose dolphin (*Tursiops spp*.) exhibit symmetrical half-power beam widths between 8 – 9° [59–61]. Wider beam widths have been observed for the harbor porpoise (*Phocoena phocoena*) with a beam width of 11° and 13° in the horizontal and vertical planes, respectively [62] up to the Ganges river dolphins (*Platanista gangetica*) with a beam width of 14.5° [63]. Ganges river dolphins inhabit shallow, riverine waters that may explain their wider sonar beam when compared to pelagic species [63]. In contrast to species with restricted distributions like the narwhal [64], belugas occupy a diverse range of habitats given their circumpolar distribution in the Arctic and sub-Arctic [1, 13–15] which may result in greater sonar variability across subpopulations. Here we show belugas occupying the deep, ice-covered waters in Baffin Bay present clicks with high directionality (DI > 30 dB), high SLs, and ICIs of ~100 ms which all facilitate long-range detection and spatial filtering. Environments like riverine or coastal systems with vegetation and shallow depths can create conditions with high acoustic clutter and reverberation levels when compared to open ocean conditions [57, 63]. Narrow, short-range biosonar systems adapt best to riverine environments to reduce reverberation and clutter. It may be that beluga subpopulations that predominantly inhabit rivers or coastal habitats present clicks with characteristics like the Ganges or Amazon river dolphins with lower SLs (<200 dB re 1 μPa_pp_), a slightly wider beam (>10°), and shorter ICIs (<50 ms) to adapt to their environment [63]. Future work to establish estimates of sonar parameters from several wild beluga stocks (e.g., Cook Inlet, Alaska [32, 35, 65] or the Canadian Arctic [22, 66]) will demonstrate whether characteristics of beluga biosonar including a narrow beam and high SLs remain consistent across diverging habitats.

As the only members of the Monodontid family, belugas and narwhals have broadband, high-frequency clicks and a narrow beam width, sonar properties that are broadly shared with other echolocating delphinids [48, 67]. Inverse frequency scaling where larger animals vocalize at higher source levels and lower sound frequencies compared to smaller animals has been an accepted hypothesis in acoustic communication for both terrestrial and marine environments [68, 69]. Jensen et al. [48] tested this hypothesis by claiming that this law operates differently for echolocating toothed whales. They examined how source level, directionality, and frequency vary with body size and reported that sonar output increased with body size at twice the rate than expected. As a result, having a narrow acoustic field of view, consistent for all toothed whales, is likely to be the primary evolutionary pressure for odontocete biosonar [48, 62]. However, among toothed whales with average -3 dB beam widths between 5 to 14°, belugas and narwhals are outliers with both high source levels and narrow beam widths relative to their body size (Figs 2A and 3D in Jensen et al. [48]). This suggests that although many delphinids share common acoustic properties, Arctic odontocetes may have specific biosonar adaptations to increase their detection range, decrease surface reflections in their ice-dominated environment, and effectively navigate through leads in the pack ice. In dense pack ice conditions, belugas and narwhals rely on openings in the ice to breathe at the surface, so there is a strong selective pressure to locate open water and ensure survival. Our results corroborate the notion that Arctic odontocetes are outliers among all toothed whales by having the narrowest acoustic field of view.

Echolocation is characterized by highly-directional acoustic emissions where signals become increasingly distorted off-axis to the center of the beam [38, 42, 70, 71]. As a result, accurate sonar parameter estimates are contingent on the ability to isolate on-axis clicks. While the criteria used in this study to isolate on-axis clicks (Fig 2) follows similar studies [46, 55–58], the true acoustic axis from which the echolocation signals originated remains unknown. However, the beam width measurement reported here closely compares to that of Au et al. [25] where the acoustic axis was known. Furthermore, examination of signals recorded on receivers away from the maximal RL—assumed to be the center of the beam—show signal distortions (Fig 6). Finally, when comparing spectra between the 133 vertical on-axis clicks to the 12 horizontal and vertical on-axis clicks, it is apparent that high frequency content is lost as horizontal off-axis clicks are introduced in the sample (Fig 5A and 5C). The 12 on-axis clicks isolated in this study retain high-frequency content and reveal a narrow beam width consistent with captive experiment estimates, indicating they are likely close to the whale’s acoustic axis.

Au et al. [25] reported highly variable and multilobed vertical beam patterns for low-amplitude clicks and for sequences where click intervals were less than 5 milliseconds. The authors further describe that the major axis of the composite vertical beam pattern was elevated 5° above the horizon, defined by the plane of the animal’s teeth [25]. Due to the *in situ* recording environment of the data analyzed here, the orientation of the animal’s cranium and body was unknown. Moreover, identifying lobes in the acoustic beam pattern with any certainty was impossible due to the spatial arrangement of the receivers; the recording aperture was too wide to yield a sufficient resolution to capture lobes. Yet, our results show an asymmetrical vertical beam width with a wider ventral beam, providing further evidence for beluga biosonar adaptability and evolutionary adaptations in the Arctic environment. A sonar beam with a wider ventral beam more effectively filters surface clutter from the pack ice when compared to a symmetrical beam. It is possible that Au et al. [25] observed a symmetrical sonar beam given the captive environment devoid of dense sea ice, whereas the free-ranging beluga beam width estimates reported here show context-specific biosonar adaptability in response to the ice-dominated setting.

Using the sonar equation and assuming a noise limited environment with spherical spreading, we computed a theoretical detection range for two scenarios: the maximum range for a beluga to ensonify a target prey and the maximum distance for a beluga click to reach an acoustic receiver. For both calculations, we used a SL of 212 dB re 1 μPa, which we computed from the 12 on-axis clicks used this study (Table 1). Transmission loss was calculated as 20log(*r*) + α*r* where *r* is the distance in meters from the source and α (absorption) is 0.03 dB/m (estimated at 100 kHz) [52]. The theoretical detection range for a beluga to ensonify a target prey, Arctic cod (*Boreogadus saida*), is approximately 300 meters using a prey target strength of -45 dB [72] and beluga hearing threshold of 50 dB re 1 μPa [73]. However, beluga hearing thresholds have been shown to vary widely (>30 dB) [73], and as a result, beluga prey detection ranges will also vary considerably. The estimated detection range for a beluga click reaching a hydrophone with a broadband system noise level of 130 dB re 1 μPa, reflecting receivers used in this study, is approximately 800 meters. Indeed, there are many factors that drive the detection range of a beluga click, but these estimates along with the knowledge that belugas have a narrow beam provide important information when determining the density and spatial range to deploy future PAM receivers.

### Patterns in frequency spectra

Beluga spectra from on-axis clicks showed a two-lobed pattern with characteristic peaks at 90 and 150 kHz and a slight notch at approximately 130 kHz that has not been previously reported in captive or wild beluga acoustic studies (Figs 5A, 5C and 6). However, the high peak frequencies for clicks on-axis in both the horizontal and vertical planes (97 kHz) and on-axis in only the vertical plane (90 kHz) are consistent with earlier work [18, 33]. It is possible that the animals used in experimental studies did not produce such high frequency, broadband clicks in the captive environment, or alternatively the sampling rate of the recording designs were too low to sufficiently capture the second peak. Nonetheless, we expect that the second peak at 150 kHz reported here was underestimated, and in reality, this peak is likely more pronounced given that higher frequencies attenuate faster than lower frequencies for broadband signals.

Apart from the bimodal frequency pattern, the broadband spectra reported here aligns with characteristic spectral patterns for *Delphinidae* species. Extant toothed whale echolocation has been classified into four main click types: 1) multi-pulsed sperm whale (*Physeteriidae*) clicks; 2) frequency modulated beaked whale (*Ziphiidae*) clicks; 3) broadband delphinid clicks; and 4) narrow-band, high frequency clicks [48]. As part of the delphinid click type, belugas produce broadband signals similar to those generated by riverine and marine delphinids (e.g. *Tursiops* and *Orcaella* genera) [42, 48, 63]. Beluga spectra reported here, however, demonstrate a broadband signal with the presence of a unique spectral lobe pattern that distinguishes it from other delphinids.

The broadband, lobed frequency pattern of beluga clicks shown in this study may provide necessary information to differentiate beluga echolocation from other species with similar acoustic profiles and spatial distributions, such as the killer whale (*Orcinus orca*) or narwhal. Increasing anecdotal and empirical evidence have showed range expansions of killer whales in the eastern Canadian Arctic and Baffin Bay, West Greenland, likely the result of declines in sea ice and increase in suitable habitat and foraging grounds [74]. Range shifts are likely as the Arctic changes, and knowledge of Arctic odontocete spectra are critical when choosing or designing recording equipment to measure echolocation parameters correctly and maximize characteristic echolocation features for species identification. When comparing killer whale and beluga spectra, broadband bimodal frequency spectra have been documented in killer whale clicks [75, 76] resembling the beluga lobed spectra we observed. Killer whale low and high frequency peaks occur at 24 and 108 kHz, respectively, [75] in contrast to the higher frequency peaks in beluga spectra reported here (97 and 147 kHz; Fig 5A). When comparing narwhal and beluga spectra, the narwhal presents a unimodal spectrum with no clear spectral notches [46]. Additionally, beluga click peak frequency (97 kHz) is higher than that of the narwhal (~70 kHz) [46, 50] which may prove useful for species classification. Following work done by Soldevilla et al. [77] where Risso’s and Pacific white-sided dolphins (*Lagenorhynchus obliquidens*) were classified using spectral properties including unique peaks and notches, our results show promise for species identification using spectral information from beluga echolocation and other odontocetes found in Arctic waters. However, particular attention to sampling rate and on-axis click criteria must be carefully considered, since the presence of defined spectral peaks diminishes with increasing off-axis angle (Fig 6) and recordings using low sampling rates may lack high frequency content.

### Scanning behavior

Biosonar scanning behavior, also referred to as shifts in acoustic gaze, has been well documented for bats in both field and laboratory settings [78–81], but it is much less described for echolocating marine mammals. Scanning is typically identified using a hydrophone array where successive changes in the maximal RL on individual receivers throughout a click train are measured, revealing movement of the animal’s sonar beam over time. Scanning of echolocating marine mammals was first reported by Schevill and Lawrence [82] and later by Kellogg [83] and Norris et al. [84], though all accounts were largely qualitative descriptions for captive experiments with bottlenose dolphins (*Tursiops truncatus*). Recent studies with captive harbor porpoises (*Phocoena phocoena*) describe scanning behavior and speculate that it increases the animal’s sensory volume, exploiting sonar beam directionality [45, 47]. Changes in an animal’s acoustic gaze may also function to assist in moving target localization. Kloepper et al. [85] showed one captive bottlenose dolphin directed the center of its beam slightly off-axis to its target and concluded that the animal’s off-axis emission strategy maximized angular position estimates of the target. In contrast, Beedholm et al. [49] gave two trained delphinids (*Tursiops truncatus* and *Pseudorca crassidens*) the same detection task and reported that while they rarely positioned their beam’s axis directly on the target, in each trial the target was ensonified within the animal’s half-power beam width, likely to increase echo-to-noise ratios. A growing body of evidence has demonstrated the various ways animals adjust their acoustic gaze, including changes in beam width when approaching a target [37, 45]. Further research is needed to investigate potential differences in scanning behavior among delphinid species, examining whether directionality and scanning angle are inversely correlated or behavioral variations exist between coastal and marine environments. Nonetheless, vertical scanning was reported for narwhals by Koblitz et al. [46], and we similarly show this behavior for belugas (Fig 7). To our knowledge, our results mark the first account to quantify scanning behavior of belugas and support the assertion that scanning is a compensatory trait that widens the whale’s acoustic gaze in response to a highly directional sonar beam. The degree to which wild beluga beam width changes during target selection remains to be studied.

### Error estimation and limitations

Localization error was determined using methods and results outlined in Koblitz et al. [46] and is consistent with other studies using linear arrays for localization [56, 58, 63, 86]. Beluga recordings analyzed here were from the same field season and used the same instruments as Koblitz et al. [46] so error estimation equally applies. Accordingly, the source distance is expected to be underestimated by approximately 20%, resulting in an underestimation of the ASL by 1 to 4 dB. The beam width is likely overestimated as it is inversely proportional to the localized distance; further, our methods to account for any fluctuations in individual hydrophone sensitivities by interpolating and smoothing beam patterns resulted in a conservative vertical beam width estimate. When considering the distances that whales were recorded (up to 100 meters) for this study, our vertical beam width measurement is estimated to be 1 to 2° wider than the true beluga vertical beam width. Thus, the true beluga vertical beam width is likely narrower and bears a higher directivity than what is reported here (5.4°).

Although the sample sizes used here for on-axis clicks are comparable to other biosonar studies [46, 55, 56, 58], there are limitations to the conclusions presented in this study to parameterize wild beluga echolocation given the small samples and number of individuals considered. Based on the localization analyses and individual tracks (i.e., click sequences) produced, we estimated 3 to 12 individuals produced the on-axis clicks that were used for sonar parameter calculations (12 on-axis clicks in both the horizontal and vertical planes; 133 clicks on-axis in the vertical plane). These data likely originated from the same group of individuals, which indicates that any potential variation in echolocation characteristics across different groups or stocks would not be represented in the parameter estimates presented here. Yet, the parameters reported here provide a strong foundation on which future work will continue to refine. In particular, continued research is needed to quantify how beluga echolocation varies across subpopulations that inhabit a variety of environments and how it broadly compares to odontocete echolocation in and outside of the Arctic.

### Implications for PAM in a changing Arctic

An increase in human activities and ambient underwater sound levels pose significant risks to Arctic cetaceans as warming temperatures lead to sea ice loss and a longer open-water season [1, 2]. Anthropogenic noise (e.g. commercial ships, pile drivers, helicopters, outboard motors) has been shown to mask beluga communication and hearing for the Cook Inlet population [9] and could similarly threaten populations in West Greenland. Human expansion northward into the Arctic is expected for oil and gas exploration, fishing, and recreation as regions like the Northwest Passage become ice-free and navigable. In order to develop management plans and regulations that proactively minimize threats to Arctic cetaceans, monitoring programs that provide baseline data for cetacean ecology and their habitats are needed.

PAM that uses multiple hydrophones over large spatiotemporal scales is the most viable tool to study Arctic cetacean populations in remote regions and identify ecologically important areas for management, including migration routes and mating grounds [33, 36, 87–90]. Our results provide fundamental sonar parameters for belugas that can be implemented in Arctic and sub-Arctic PAM programs, particularly for the comparison and differentiation between belugas and narwhals. We also provide an approximation of 800 m as the range at which a beluga click can be detected using a PAM receiver similar to the instruments used in this study. Understanding the spatial resolution at which whales can be detected is important when designing the extent and frequency of PAM receivers to be deployed. Classification using echolocation is a promising tool, given its primary role in beluga and narwhal foraging ecology, and is increasingly being incorporated into classification regimes [77, 91, 92]. To fully implement PAM using echolocation identifiers, appropriate acoustic receivers with a high sampling rate must be used. Arctic odontocetes use echolocation frequently as it is the primary way they sense their environment and locate prey, and as a result, echolocation data are especially useful for classification during times where vocalizations (e.g., whistles) are absent. This *in situ* study of beluga biosonar provides foundational information for PAM programs and a baseline for future comparative studies with species that have overlapping ranges and similar acoustic profiles.
